# A case of acute macular neuroretinopathy in a pediatric patient with optic neuritis

**DOI:** 10.1016/j.ajoc.2025.102332

**Published:** 2025-04-10

**Authors:** Ami Nakagawa, Sujin Hoshi, Tetsuro Oshika

**Affiliations:** Department of Ophthalmology, Institute of Medicine, University of Tsukuba, 1-1-1 Tennoudai, Tsukuba City, Ibaraki, Japan

**Keywords:** Acute macular neuroretinopathy, Optic neuritis, Myelin oligodendrocyte glycoprotein antibody-associated disease, Optical coherence tomography, Pediatric patient

## Abstract

**Introduction:**

Acute macular neuroretinopathy (AMN) is a retinal disorder caused by ischemia in the deep capillary plexus. There have been several reports of AMN associated with optic neuritis in adults. We report a case of AMN associated with optic neuritis in a pediatric patient.

**Case report:**

A 14-year-old boy presented with ocular pain upon eye movement and decreased vision with disc edema in the right eye, leading to a diagnosis of optic neuritis. After initiating steroid pulse therapy, his visual acuity improved promptly. Optical coherence tomography revealed a hyperreflective area in the outer retina on the nasal side of the macula, and fundus examination showed a reddish-brown, wedge-shaped lesion, confirming the diagnosis of AMN. The case was ultimately diagnosed as myelin oligodendrocyte glycoprotein (MOG) antibody-associated disease. Post-treatment, visual acuity improved; however, a paracentral scotoma attributed to AMN remained.

**Discussion and conclusion:**

The pathogenesis of AMN in cases of optic neuritis may involve mechanical arterial occlusion due to optic disc swelling. Regardless of age, careful fundus examination is necessary during the clinical course of optic neuritis, and MOG-immunoglobulin G testing can be considered for patients showing optic neuritis with AMN.

## 1 Background

Acute macular neuroretinopathy (AMN) is a relatively rare condition characterized by reddish-brown, wedge-shaped lesions within the retina. These lesions are often associated with mild vision loss and paracentral scotoma.^1^ In the early stages, optical coherence tomography (OCT) reveals hyperreflective lesions in the inner and outer retinal layers, with subsequent thinning observed in later stages.^2^ AMN has been reported to occur in conjunction with optic neuritis in adults^3^; however, no such cases have been reported in pediatric patients. Here, we report the case of a boy who developed AMN during treatment for optic neuritis.

## 2 Case presentation

**Fig. 1:**
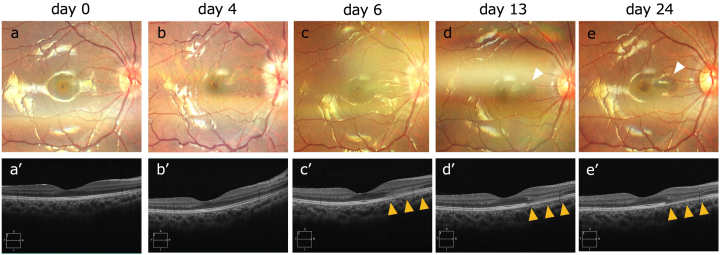
Clinical and imaging findings for a 14-year-old Japanese boy with acute macular neuroretinopathy associated with optic neuritis. The patient presented with pain in the right eye during eye movements. On the day of presentation (day 0), fundoscopy revealed optic disc swelling (a), while OCT revealed a normal macula (a’). Four days after presentation (day 4), the BCVA in the right eye decreased to 20/25, and fundoscopy reveals worsening optic disc swelling and tortuosity of the retinal arteries (b), while OCT reveals no change in the macula (b’). Six days after presentation (day 6), the disc swelling has reduced (c), but OCT shows hyperreflectivity of the outer nuclear layer on the nasal side of the macula in the right eye (c’, yellow arrowhead). Thirteen days after presentation (day 13), fundus examination reveals a reddish-brown, wedge-shaped lesion (d, white arrowhead), while OCT shows thinning of the outer nuclear layer (d’). Twenty-four days after presentation (day 24), the wedge-shaped lesion in the fundus remains unchanged (e), and OCT shows further thinning of the nasal outer retinal layer (e’). BCVA = best-corrected visual acuity, OCT = optical coherence tomography.

**Fig. 2:**
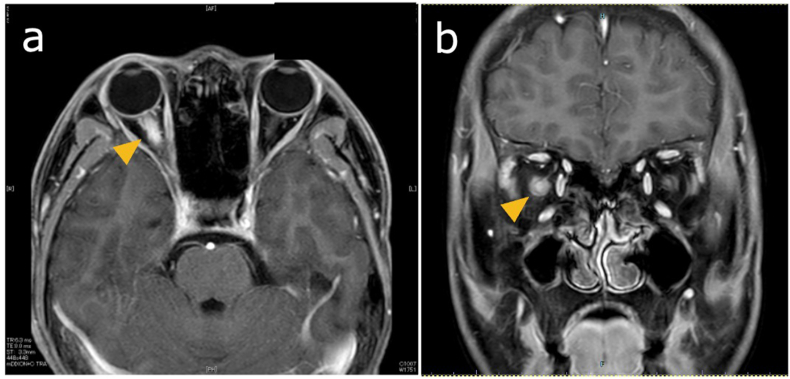
Magnetic resonance imaging (MRI) findings for a 14-year-old Japanese boy with acute macular neuroretinopathy associated with optic neuritis. MRI of the orbits shows swelling and enhancement of the right optic nerve and perineural space (a: axial, b: coronal).

**Fig. 3:**
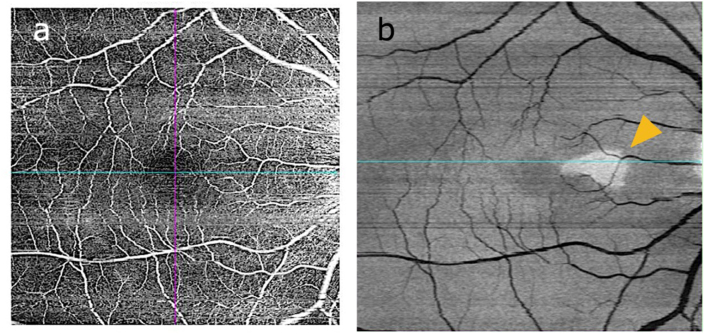
Optical coherence tomography (OCT) findings after the first course of treatment for a 14-year-old Japanese boy with acute macular neuroretinopathy associated with optic neuritis. The patient was administered intravenous methylprednisolone (1000 mg daily for 3 days). The best-corrected visual acuity in the right eye improved after treatment. OCT angiography reveals normal findings (a), and en face OCT shows enhancement in the outer retinal (b).

A 14-year-old Japanese boy presented at our ophthalmology clinic with pain in the right eye during eye movements for 7 days. His best-corrected visual acuity (BCVA) was 20/16 in both eyes, and the critical flicker fusion frequency was 40 Hz in both eyes. A relative afferent pupillary defect was observed in the right eye, which also showed optic disc swelling on fundoscopy. OCT revealed a normal macula (Fig. 1a and a’). Four days after presentation, the BCVA in the right eye decreased to 20/25, and fundoscopy revealed worsening optic disc swelling with tortuosity of the retinal arteries. OCT revealed no change in the macula (Fig. 1b and b’). Magnetic resonance imaging (MRI) of the orbits revealed swelling and enhancement of the right optic nerve and perineural space (Fig. 2a and b), confirming optic neuritis. The patient was administered intravenous methylprednisolone (1000 mg daily for 3 days). Six days after presentation, the BCVA in the right eye improved to 20/16, and the disc swelling reduced (Fig. 1c). However, OCT revealed hyperreflectivity of the outer nuclear layer on the nasal side of the macula in the right eye (Fig. 1c’). Thirteen days after presentation, OCT indicated thinning of the outer nuclear layer, and fundus examination revealed a reddish-brown, wedge-shaped lesion (Fig. 1d and d’). OCT angiography also clearly depicted the lesion, with enhancement in the outer retinal layers (Fig. 3a and b). Based on these findings, the patient was diagnosed with AMN.

Following treatment initiation, the patient's vision initially showed rapid improvement; however, when the steroid dose was reduced, it worsened. Therefore, at 16 days after presentation, the patient received a second course of intravenous methylprednisolone (1000 mg daily for 3 days), following which his vision improved again, allowing gradual reduction of prednisolone by the oral route (tapered by 5 mg every week). Serum analysis performed before the second steroid pulse revealed negative results for anti-aquaporin-4 and anti-myelin oligodendrocyte glycoprotein (MOG) antibodies, as confirmed by use of a live cell-based assay.

**Fig. 4:**
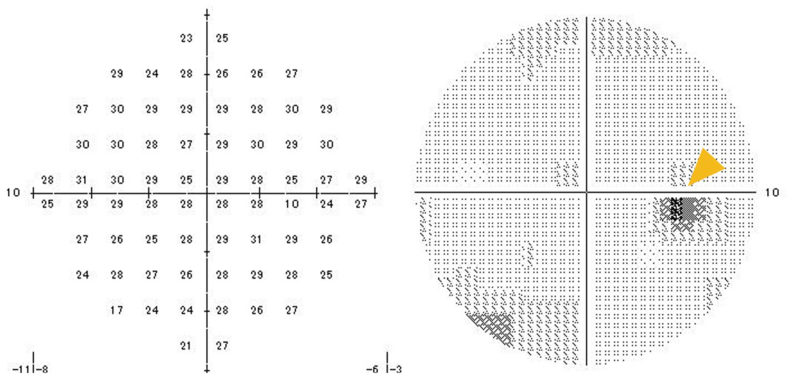
Static perimetry findings after the second course of treatment for a 14-year-old Japanese boy with acute macular neuroretinopathy associated with optic neuritis. At 16 days after presentation, the patient received a second course of intravenous methylprednisolone (1000 mg daily for 3 days). At 24 days after presentation, static perimetry reveals paracentral scotomas. Static perimetry was performed using the Humphrey Field Analyzer Swedish Interactive Thresholding Algorithm (SITA) Fast 10-2 protocol. The reliability indices indicated a 0 % false-positive rate and an 11 % false-negative rate.

By 24 days after presentation, the wedge-shaped lesion in the fundus remained unchanged, and OCT indicated more thinning of the nasal outer retinal layers (Fig. 1e and e’). Static perimetry revealed paracentral scotomas corresponding to the lesion (Fig. 4). No further deterioration in visual acuity or fundus findings were observed thereafter.

**Fig. 5:**
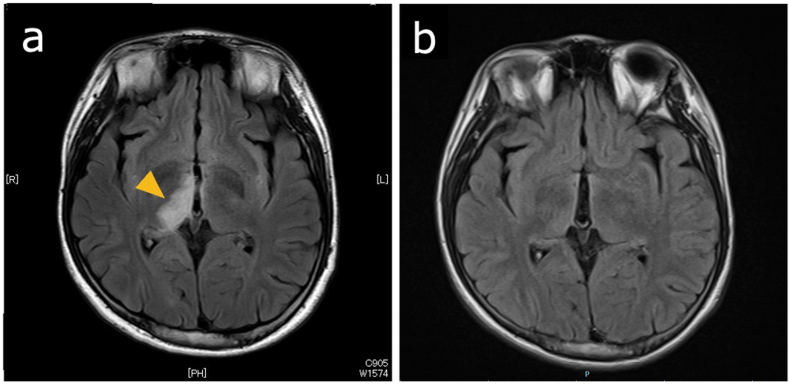
Magnetic resonance imaging (MRI) findings before and after diagnosis and treatment for myelin oligodendrocyte glycoprotein antibody-associated disease in a 14-year-old Japanese boy with acute macular neuroretinopathy associated with optic neuritis. Brain MRI reveals demyelinating lesions extending from the right thalamus to the midbrain (a). After treatment with two courses of intravenous methylprednisolone (1000 mg daily for 3 days), seven plasma exchange sessions, one intravenous immunoglobulin treatment session, and azathioprine, follow-up MRI shows reduced demyelinating lesions (b).

At 53 days after presentation (while on prednisolone 40 mg per day by the oral route), the patient developed coronavirus disease 2019. At 63 days (while on prednisolone 35 mg per day by the oral route), he developed diplopia, and at 70 days (while on prednisolone 30 mg per day by the oral route), he experienced an abnormal sensation in the left upper and lower limbs along with somnolence. Brain MRI revealed demyelinating lesions extending from the right thalamus to the midbrain (Fig. 5a). Even after two more courses (a total of 4 courses) of intravenous methylprednisolone (1000 mg daily for 3 days), his symptoms did not alleviate, and he underwent seven plasma exchange sessions and one intravenous immunoglobulin (Ig) treatment session. Due to the refractory nature of this case, Azathioprine was also initiated. Subsequently, his symptoms alleviated, and follow-up MRI showed reduced demyelinating lesions (Fig. 5b). Cerebrospinal fluid (collected on day 70) analysis using a live cell-based assay revealed positivity for anti-MOG antibodies. On the basis of this finding as well as imaging findings, the patient was diagnosed with MOG antibody-associated disease (MOGAD).

## 3 Discussion

Bos and Deutman first described AMN in 1975.^4^ The most common triggers of AMN include viral infections and vaccinations, followed by the use of oral contraceptives. Other factors include the use of sympathomimetic drugs, trauma, and dehydration.^1^ Recently, cases of AMN following coronavirus disease 2019 and vaccination have also been reported.^5^ AMN is thought to result from ischemia of the deep capillary plexus, which leads to damage to the outer retina. Early OCT findings include hyperreflectivity in the outer plexiform and outer nuclear layers, followed by thinning of the outer nuclear layer and disruption of the ellipsoid zone.^2^ The outer plexiform and nuclear layers are located at the boundary between the retinal and choroidal circulation, making them susceptible to ischemic changes in cases of circulatory insufficiency.^1^

A report of optic neuritis noted that six of 114 patients with acute optic neuritis also had AMN.^3^ All six cases involved adults aged 26–56 years, and we found no prior reports of such cases in a literature search of the PubMed and Google Scholar databases using the keywords *acute macular neuroretinopathy*, *optic neuritis*, and *child* in November 2024. The six patients in the previous report included three with anti-MOG antibody-positive optic neuritis, two with multiple sclerosis, and one with idiopathic optic neuritis. Chen et al.^6^ have reported a correlation between the severity of optic disc swelling and decreased peripapillary blood flow and that inflammatory edema of the optic nerve may compress the retinal arteries and reduce the blood flow in the deep capillary plexus. Feucht et al.^7^ also reported reduced vessel density in the superficial and deep capillary plexuses on OCT angiography in patients with multiple sclerosis and optic neuritis. Reports have documented retinal ischemic disorders such as paracentral acute middle maculopathy and retinal artery occlusion associated with MOGAD.^8^, ^9^, ^10^ The characteristic anterior optic nerve involvement in MOGAD^11^ is thought to lead to optic disc edema, which subsequently compresses the retinal vascular plexus, resulting in impaired blood flow. A similar mechanism is hypothesized to underlie the development of AMN. In this case, AMN was not initially present at the onset of optic neuritis, it developed shortly after the worsening of optic disc edema. During the further deterioration of optic disc swelling, tortuosity of the retinal arteries was observed (Fig. 1c), suggesting that the retinal arteries running through the lamina cribrosa may have been compressed. Additionally, OCT angiography revealed decreased vessel density in the superficial and deep capillary plexuses. These findings suggest that the development of AMN was caused by mechanical compression of the retinal vasculature due to the aforementioned papilledema.

Although rare, the possibility of AMN development during optic neuritis should be considered, even in pediatric patients, and careful funduscopic monitoring is warranted throughout the clinical course of the disease. In addition, MOG-IgG testing can be considered, including cerebrospinal fluid when negative in the serum as it can potentially diagnose nearly 10 % of MOGADs that are negative in the serum^12^^,^^13^ for patients showing optic neuritis with AMN.

## CRediT authorship contribution statement

**Ami Nakagawa:** Writing – original draft, Investigation, Data curation. **Sujin Hoshi:** Writing – review & editing, Conceptualization. **Tetsuro Oshika:** Writing – review & editing, Supervision.

## Patient consent

The patient provided written informed consented for publication of this case report and accompanying images.

## Claim of priority

We found no prior reports of such cases after conducting a literature review in November 2024 utilizing PubMed and Google Scholar using the keywords AMN, optic neuritis, and child.

## Authorship

All authors attest that they meet the current ICMJE criteria for authorship.

## Funding

This research did not receive any specific grant from funding agencies in the public, commercial, or not-for-profit sectors.

## Declaration of competing interest

None.
